# Road to FAIR genomes: a gap analysis of NGS data generation and sharing in the Netherlands

**DOI:** 10.1136/bmjos-2021-100268

**Published:** 2022-04-12

**Authors:** Jeroen A M Belien, Anke E Kip, Morris A Swertz

**Affiliations:** 1 Pathology, Amsterdam UMC, Vrije Universiteit Amsterdam, Amsterdam, The Netherlands; 2 Lygature, Utrecht, The Netherlands; 3 Genetics, UMCG, Groningen, The Netherlands

**Keywords:** translational medical research, informatics

## Abstract

**Objective:**

This study investigates current standards and operational gaps in the management and sharing of next generation sequencing (NGS) data within the healthcare and research setting and according to Findable, Accessible, Interoperable and Reusable (FAIR) principles.

**Methods:**

The analysis was performed as the basis from which to bridge identified gaps and develop widely accepted working standards that ensure optimal reusability of genomic data in healthcare and research settings in the Netherlands. This work is part of the ‘Rational Pharmacotherapy Program’ led by ZonMw, The Netherlands Organisation for Health Research and Development, which aims to promote the efficient implementation of NGS and personalised medicine within Dutch healthcare, with an initial focus on oncology and rare diseases.

**Results:**

Based on this analysis and as part of this programme, a consortium was formed to develop an instruction manual for FAIR genomic data in clinical care and research based on an inventory of commonly used workflows and standards in the (inter)national field of genome analysis.

**Conclusions:**

The gap analysis presented and discussed in this paper represents the starting point for this inventory and is a possible contribution from the Netherlands to the European 1+ Million Genomes Initiative. This paper addresses the topics of data generation, data quality, (meta)data standards, data storage and archiving and data integration and exchange.

**Strengths and limitations of this study**

- This study provides insight in current standards and operational gaps in the management and sharing of next generation sequencing (NGS) data within the healthcare and research setting and according to Findable, Accessible, Interoperable and Reusable principles in the Netherlands.
- The methods and materials used can be used by other countries to determine their maturity with regard to management and sharing of NGS data.
- In-depth interviews have been conducted with many different stakeholders and organisations.
- The study has been conducted in the Netherlands, some findings/processes might therefore be specific to the Netherlands.

## Introduction

The aim of the Rational Pharmacotherapy Program (Goed Gebruik Geneesmiddelen; GGG) of ZonMw (The Netherlands Organisation for Health Research and Development, https://www.zonmw.nl/en/) is more efficient, safe and suitable use of existing medicines. Within the GGG programme, the Personalised Medicine (PM) programme intends to make structural contributions to a future healthcare system in which each patient can count on tailored therapy based on their individual characteristics. The aim of the PM is to ensure efficient implementation of next generation sequencing (NGS) and personalised medicine in the Dutch healthcare system, with an initial focus on oncology and rare diseases. The programme stimulates collaboration, facilitates the use of process standards and studies the cost-effectiveness of diagnostic tools and pharmacotherapy.

In 2013, a group of Dutch experts, including pathologists, medical oncologists, geneticists and ethicists from healthcare and research backgrounds, and the National Health Care Institute of the Netherlands compiled an internal report (unpublished but available from ZonMw on request) that addressed the challenges and issues posed by the implementation of NGS and personalised medicine in Dutch healthcare. In short, the report stated that genome analysis—especially NGS—brings with it specific challenges with respect to data, ethics, financing and information technology. In addition, modern genetic research questions involve large data sets that are often distributed over different laboratories and departments. This work therefore requires the integration of genotype and phenotype data captured in different systems, making it essential to standardise the storage, sharing and analysis of DNA and phenotype data across research facilities and care units in the Netherlands in order to ensure optimal reusability of data while also adhering to appropriate levels of security and privacy standards.

Addressing these challenges is part of the ZonMw PM research programme. Within this programme, the data management working party has formulated the following overall objective—*to ensure optimal reusability of genomic data according to FAIR principles both in the healthcare setting and for future research*—where FAIR stands for ‘Findable, Accessible, Interoperable and Reusable’ and can be considered an international guideline for high-quality data stewardship.^1–3^

In the first quarter of 2018, based on the stated objective and developments within the fields of FAIR data usage and NGS, ZonMw ordered an analysis investigating current standards and gaps in optimal data management of NGS data according to FAIR principles, including the topics of data generation, data quality and (meta)data standards, data storage and archiving, data integration and exchange.^4^ This paper outlines the subsequent analysis, its results and possible next steps, all of which are being used as the starting point for a ZonMw open call^5^ to set up a consortium to develop an instruction manual for FAIR genomic data in clinical care and research.

## Methods

### Outline of the gap analysis

In this study, the gap analysis is defined as the process that allows stakeholders to determine how to best achieve data management and sharing of NGS data. It compares the current state with an ideal state or goals, which highlights shortcomings and opportunities for improvement.

To obtain a complete picture of the current state of affairs with respect to standards and gaps in optimal data management of next generation genome sequencing data according to the FAIR principles in the Netherlands, and supported by as many stakeholders as possible, we have taken the following steps (see also online supplemental figure 1 shared on Zenodo (https://doi.org/10.5281/zenodo.6305055)). First, we produced a generic NGS process diagram, based on a commonly used care workflow, that could be used to support the subsequent interviews (step 1). As research often focuses on one or more subparts of this diagram, the diagram is also applicable to research. We then drafted a questionnaire about the inventory of (meta)data standards and retrieval of gaps (step 2), which we used as a basis for the subsequent interviews with diverse experts and individuals involved in running all the ZonMw PM projects (step 3). In parallel with the first three steps, we performed a short literature review to identify current practice and potential future practice within NGS (step 4). We then processed the interviews and identified, anonymised and classified current gaps in data management and/or data processing in genome sequencing processes (step 5). Finally, we are now sharing the results with the community through presentations, publications and suggestions for next steps for addressing the identified gaps. Below, we detail the motivation and execution of each of these steps.

#### Step 1. A generic NGS process diagram

**Figure 1:**
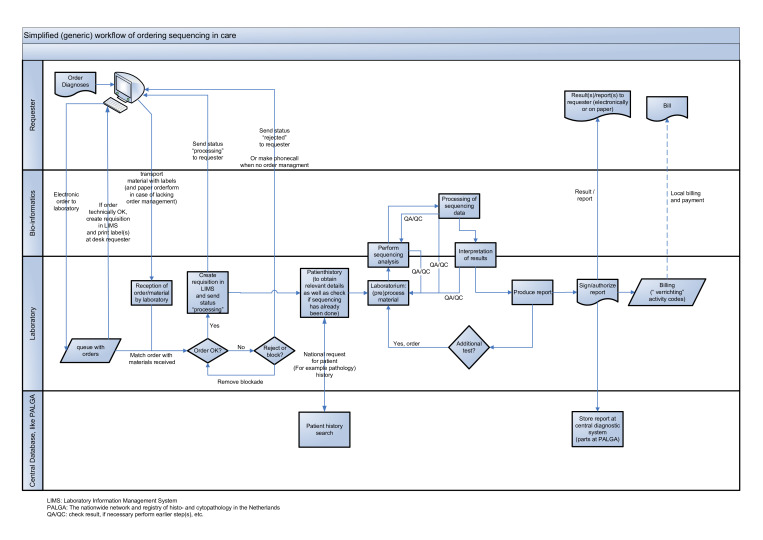
High-level, simplified generic data and workflow flow chart showing the natural order of sequential subprocesses, starting with the ordering of a next generation sequencing (NGS) test (left side of the diagram) and following through to the reporting of the test results (right side of the diagram). The diagram is divided into four lanes to indicate which participants handle which subprocesses (the blue items/forms) and shows handover moments for either materials and/or data (the connecting arrows). The participants in this process include the individual requester of the NGS analysis, the bioinformatician, the laboratory and the workflow-relevant (national) databases. LIMS, laboratory information management system; PALGA, the nationwide network and registry of histopathology and cytopathology in the Netherlands; QA/QC, check result, if necessary perform earlier step(s), etc.

To facilitate interviews, we developed a simplified NGS process diagram that follows the NGS process from the analysis request to reporting of the result in the context of a healthcare setting (figure 1). To streamline the diagram, we did not include a separate stream for research. However, as research often focuses on one or more subparts of the diagram, the diagram can also be used to describe the NGS process in research.

The simplified flow diagram shows the natural order of sequential subprocesses, starting with the ordering of an NGS test (figure 1, left side of the diagram) and following through to the reporting of the test results (figure 1, right side of the diagram). The diagram is divided into four lanes to indicate which participants handle which subprocesses (figure 1, blue items/forms) and shows handover moments for either materials and/or data (figure 1, connecting arrows). The participants in this process include the individual requester of the NGS analysis, the bioinformatician, the laboratory and the workflow-relevant (national) databases.

The process starts when the treating physician (requester) orders a diagnostic NGS test for a patient, either digitally or on paper. If ordered digitally, the order is received by the laboratory information system and, if deemed technically sound, labels to identify the patient material to be analysed are printed at the site of the requester. If ordered on paper, the requester manually puts the mandatory identifier(s) on the material. Next, the labelled material is transported to the laboratory, along with the paper order (in case of a request on paper). Once the laboratory receives the order and material, it matches the order with the material received. If the order is okay, a requisition is created in the laboratory information system. If applicable, the requester will be sent a status message stating that the requested order is being processed. If there is an issue with the order or the accompanying material, the laboratory either (1) places the order on hold (a block), if the issue identified is expected to be resolved, or (2) rejects the order and informs the requester of the reason for rejection. When available and/or relevant, the laboratory next performs a patient history search. Ideally, if an individual’s germline or tumour has already been sequenced, a laboratory specialist should check if resequencing is necessary. Once a decision has been made to sequence the received material, the material is (pre)processed so that it can be analysed by the sequencing equipment. The sequencer equipment produces sequencing data that are processed by specific algorithms under management of (primarily) bioinformaticians, and the results are transformed for interpretation by the laboratory specialist (or in case of research, by the researcher). By applying various quality assurance and/or quality control steps, a feedback process to improve and guarantee the required quality can be activated.

The interpretation of the sequencing data results in a report that, once authorised, is made available to the requester of the NGS test. The result will/can be archived in the local laboratory information management system and/or a national archive. Once the order has been completed/closed, financial aspects can proceed (eg, billing).

#### Steps 2 and 3. Questionnaires and interviews with stakeholders to inventory (meta)data standards and retrieve gaps

To facilitate the subsequent interviews, we created a questionnaire about the inventory of (meta)data standards and retrieval of gaps. This questionnaire was given to interviewees, who were asked to complete the questionnaire as completely as possible and to return it to the interviewers in time to prepare for the interview. The original questionnaire was in Dutch, but an English translation can be found in online supplemental file S1 (https://doi.org/10.5281/zenodo.6305250). Each interview was recorded and transcribed offline. Each interview was then checked/reviewed for accuracy with the interviewees and, once approved, used to extract the gaps identified during the interviews. In total, we conducted 20 interviews with 31 stakeholders, including bioinformatics experts, clinical geneticists and diagnostic experts (ie, wet laboratory experts) from different university medical centres; researchers from the ZonMw PM research projects; representatives of PALGA (the nationwide network and registry of histopathology and cytopathology in the Netherlands), the Ethical, Legal and Social Implications (ELSI) service desk, the Hartwig Medical Foundation (see https://www.hartwigmedicalfoundation.nl/en/) and the general Health Technology Assessment (HTA) and data management experts.

#### Step 4. Short literature review

We performed a quick search of the existing literature in January 2018 to identify existing standards that have been applied or are suited for application within the field of next generation genome sequencing. The search parameters used were: ‘guidelines’ AND ‘diagnostic’ AND (‘NGS’ OR ‘next-generation sequencing’) (https://pubmed.ncbi.nlm.nih.gov/?term=guidelines+AND+diagnostic+AND+(+%22NGS%22+OR+%22next-generation+sequencing%22). This search yielded 236 hits, but most of this literature provides variant analysis guidelines that are useful in a specific setting but difficult to apply to all genes in different laboratory settings.

The three most suitable PubMed matches were Richards *et al*,^6^ Matthijs *et al* 7 and Jennings *et al*,^8^ as these three articles provided more general guidelines for NGS of genetic disorders^6 7^ and somatic alterations.^8^ Richards *et al*’s^6^ article provides a revision of the standards and guidelines for the interpretation of sequence variants in all Mendelian genes, including the use of standard terminology and uniform nomenclature. Matthijs *et al*’s^7^ article provides guidelines for the evaluation and validation of NGS applications for the diagnosis of genetic disorders. Jennings *et al*’s^8^ article provides consensus recommendations for the validation and ongoing monitoring of targeted NGS panels and their diagnostic use in solid tumours and haematological malignancies. This guideline covers a broad spectrum of topics, including an NGS platform overview, test design, potential sources of error during the NGS assay development process, the optimal number of samples for validation, how to establish the minimal depth of sequencing and implementation and quality control metrics. Similar to Jennings *et al*,^8^ a fourth article by Deans *et al* 9 provides guidelines for establishing consensus standards for somatic diagnostic testing, specifically for identifying and reporting mutations in solid tumours.

We then searched the same literature for combinations of the previously used terms along with ‘data management’ or ‘FAIR’ and found four more relevant hits: Roy *et al*’s^10^ article which provides standards and guidelines for validating NGS bioinformatics pipelines; Boeckhout *et al* 11 on the FAIR-ness of guidelines for data stewardship; Sénécal *et al* 12 on legal approaches and implications with respect to NGS; and Weiss *et al* 13 on best practice guidelines for the use of NGS applications in genome diagnostics within Dutch genome diagnostic laboratories.

In addition to PubMed, we also investigated the current available standards by looking at sources like ISO (eg, https://www.iso.org/committee/4514241.html), BioPortal (https://bioportal.bioontology.org/ontologies/), The OBO Foundry (http://www.obofoundry.org/; eg, EDAM, ICO) and the Unified Medical Language System (UMLS; https://www.nlm.nih.gov/research/umls/index.html).

Relevant information from this short literature search and online review was incorporated into the questionnaire and/or used during the interviews to help identify the current state of affairs for standards and gaps in optimal data management of next generation genome sequencing data according to the FAIR principles in the Netherlands.

#### Step 5. Processing interviews and identifying, anonymising and classifying gaps

During the interviews and the processing of the interviews, all possible gaps were identified per interview. These gaps were shared with the interviewees for review, and the resulting gaps per interview were integrated into a complete list. Next, we merged overlapping gaps, and all gaps were checked for anonymity and further anonymised as needed. Each of the gaps was assigned a classification label in order to group them. Finally, the classified gaps were translated into English to facilitate the sharing of the results with the international community.

## Results

Based on the findings from the interviews, we produced (1) an updated NGS process diagram and (2) a list of 128 identified gaps.

### The updated NGS process diagram

**Figure 2:**
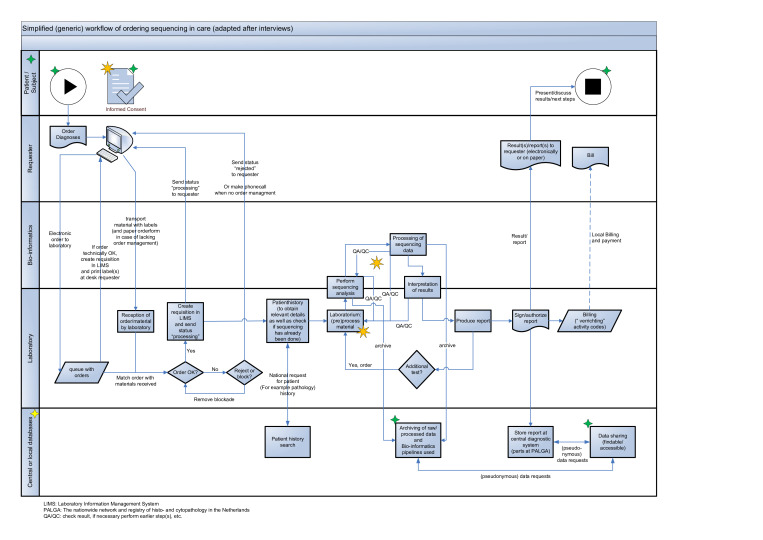
An updated next generation sequencing (NGS) process diagram (figure 1) based on the interviews carried in this analysis: (1) a separate lane has been added to detail the interaction between the patient/study subject and the analysis requester (2) the database lane has been updated to show both central and local databases, and (3) the description of the various steps has been changed to also fit NGS data generated in research. Examples of possible starting points for research have been added to the NGS process diagram as 8-pointed orange stars, but we have not added data flow arrows for several possible research (sub)questions to avoid overcomplicating the process diagram. The 4-pointed yellow (indicating a change) and green stars (indicating a new item) have been added to the diagram to more easily identify the modifications compared with figure 1. LIMS, laboratory information management system; PALGA, the nationwide network and registry of histopathology and cytopathology; QA/QC, check result, if necessary perform earlier step(s), etc.

Based on the interview results, we made the following modifications to the NGS process diagram (figure 2): (1) we added a separate lane to detail the interaction between the patient/study subject and the analysis requester (2) we updated the database lane to show both central and local databases, and (3) we changed the description of the various steps to also fit NGS data generated in research.

#### The new patient/study subject lane

The patient is the start and stop point from the care side. The patient presents a question to the doctor, who can then order test(s) to help establish a diagnosis. The results of the test(s) will be received by the doctor (the requester) and presented, explained and discussed with the patient, along with any possible next steps. In addition, we have added informed consent to the patient/study subject lane to facilitate/initiate the process of getting (a specific) permission, for example, when a healthcare provider needs to ask a patient to consent to receive therapy before providing it or when a (clinical) researcher asks a potential research participant for consent to enrol them in a clinical trial or population cohort study.

#### The updated central or local database lane

To arrive at FAIR data management in genome sequencing, the lane for central or local databases has been extended by adding subprocesses for archiving raw and/or processed data, subprocesses for archiving the computational bioinformatics pipelines applied and subprocesses for data sharing. Although the process starts and ends with the patient in the healthcare context, this is not necessarily the case in research. Research might be focused on one or more of the subprocesses related to genome sequencing, such as new types of informed consent, the improvement of DNA yield on laboratory or better bioinformatics algorithms/pipelines. These examples of possible starting points have been added to the NGS process diagram as yellow stars, but we have not added data flow arrows for several possible research (sub)questions to avoid overcomplicating the process diagram.

### Identified and classified gaps

The resulting anonymised list of 128 gaps was presented and discussed with the Data Management Workgroup of the PM. The primary focus of this analysis was data management and/or data processing in the genome sequencing processes, and the workgroup concluded that, while all 128 gaps do have a data management and/or data processing component, 47 of them fall outside the primary scope of the working group. These 47 gaps have a strong connection and overlap with three other focus areas within the PM and have therefore been given primary classifications of either ‘Education’ (6 gaps), ‘ELSI’ (26 gaps) or ‘Methodology of determining value of predictive tests (HTA)’ (15 gaps). For these focus areas, the gaps identified in our analysis could serve as an initial exploration but do not encompass a complete gap analysis because the gaps identified in these areas also have a data management component. The remaining 81 gaps were identified to belong to the primary focus of the gap analysis on data management and/or data processing requested by ZonMw and were labelled (in short) ‘Data processing’. The primary focus labels are presented in the header of each table shared in the Zenodo online supplemental file S2 (https://doi.org/10.5281/zenodo.6305563). Note that some of the 128 gaps could be classified in more than one primary focus category, as indicated in the ‘additional classification’ column shared in the Zenodo online supplemental file S2.

For the 81 gaps classified as ‘Data processing’, an additional ‘Type of hiatus’ column was added to the table in the shared files in the Zenodo online supplemental file S2 to provide subcategories of gaps addressing a more specific subject within data management and/or data processing of next generation genome sequencing data. Lastly, we have added another column labelled ‘FAIR’ to the table as shown in the Zenodo online supplemental file S2 to relate each gap back to one or more of the individual FAIR principles where applicable.

These subcategories are *uniform work process*, *financial*, *ELSI*, *data standards*, *data sharing*, *data quality*, *data management*, *data linking*, *data archiving*, *data analysis* and *operational*, and table 1 describes the nature of each of these kinds of gaps. Providing this additional layer of classification will facilitate specific experts in the field in addressing one or more specific groups of gaps (eg, by providing funding).

**Table 1:** Sub categories of gaps identified in this analysis of the NGS process

Subcategory of gap	Description
Uniform work process	Gaps related to parts of the entire process that are not yet uniform.
Financial	One gap, also linked to HTA, about lack of standardised transaction codes.
ELSI	Specific ELSI gaps with a findability/reproducibility component that could be taken up by the working group on data management and/or data processing.
Data standards	Gaps that address a variety of issues related to standards.
Data sharing	Gaps that, if resolved, would strongly ease sharing of data.
Data quality	Gaps influencing the quality of the (sub)processes of genome sequencing.
Data management	Gaps that are part of higher level, overarching data management aspects.
Data linking	Gaps identifying issues in combining data from various sources.
Data archiving	Gaps mainly pointing to sustainable data storage and use.
Data analysis	Gaps related to analysis/interpretation of genome sequence data.
Operational	Gaps pointing to issues in the (daily) operation of genome sequencing.

ELSI, Ethical, Legal and Social Implications service desk; HTA, Health Technology Assessment; NGS, next generation sequencing.

### Examples of typical gaps

To highlight recognisable issues in data management and/or data processing in genome sequencing processes associated with specific gaps, we compiled four typical examples from the interviews.

*Example 1*: A patient visits their doctor after a sudden cardiac arrest. Based on the physical examination, phenotype and medical and family history, and after consultation with the patient, the doctor requests a cardiopanel to determine if the patient’s health issue is caused by an inherited cardiac condition. Whole genome sequencing (WGS) is performed, but the doctor only receives results/answers that are limited to the cardiopanel. However, WGS actually identifies more abnormalities that are relevant for the patient, but these cannot be included with the specific cardiopanel request, otherwise it would be classified as screening. Nonetheless, given permission by a patient/subject to examine their data for research purposes, a researcher could naively gain access to data that the doctor (applicant/requester) has not seen or be able to generate new insights using tools not yet available to diagnostics. This first example is associated with, at minimum, gaps 10, 12, 14, 15, 20, 38, 45, 74, 88, 90, 102, 103 and 109 and emphasises the need for uniform working methods, alignment of legal grounds, sharing of context, informed consent and results in relation to the original purpose.

*Example 2*: Within software, the (reference) genome is often identified with self-designed strings (eg, ‘chromosome1’) instead of a unique identifier (NC_000001 and possibly versioned NC_000001.11). When adjustments are made to one of the programmes, a problem arises further along in the analysis chain because the adjustment has not (yet) been implemented. This second example is associated with, at minimum, gaps 8, 14, 22, 26, 47, 53 and 78 and makes clear that the FAIR principles apply to data and to the software.

*Example 3:* A doctor/researcher is not able to find out if someone else has already done DNA sequencing of a patient/study participant. This third example is associated with at least gaps 34 and 35, at minimum, and makes clear that by already sharing minimal NGS-related metadata unnecessary procedures and costs might be prevented.

*Example 4:* A situation where it is unclear which parts of DNA data can be shared or (re)used, and which cannot, under the new General Data Protection Regulation (GDPR) Act (Dutch AVG) that has been enforced since the end of May 2018. Under this law, DNA (data and derivatives thereof) belongs to the special categories of personal data. This fourth example is associated with, at least, gaps 89, 94, 106 and 108 and shows that implementation and interpretation of a new law sometimes can lead to unexpected confusion.

## Conclusions

This gap analysis and the 81 identified gaps presented and discussed in this paper are the starting point for the project ‘Development of instruction manual genomic data management’ that was recently awarded by ZonMw.^4^ With respect to the other 47 gaps, the 26 ELSI-related gaps have already been presented to and discussed with the ELSI Working Group, and the steps that can be taken up in the working plan of this group are currently being addressed, while the other gaps have been shared with the respective groups and will be discussed in the near future.

Recently, the European Commission Expert Group on Turning FAIR Data into reality^14 15^ published an interim report, the ‘FAIR Data Action Plan: Interim recommendations and actions from the European Commission Expert Group on FAIR data’, which was followed by an open stakeholder consultation and by a final version published on 23 November 2018.^16^ This publication has strengthened ZonMw in their vision and concrete steps already taken, as well as inspires them, on their road towards better FAIR data stewardship.

Moreover, next to being relevant to the Dutch genomics community, the results of this work as well as outputs of the awarded ZonMw project^6^ might also be applicable and of interest to various international (genomics) communities looking to make their NGS processes FAIR-er, and the flow diagrams we provide can be a useful guide for discussions about the NGS pipeline in many other countries and contexts (eg, the European 1+ Million Genomes Initiative^17^). As vice versa the awarded project will learn from and will collaborate with others like the Global Alliance for Global Health 18 and the European Alliance for Personalised Medicine 19, as well as learn from and take into account the results from leading countries like the UK, the USA, France, Finland and Australia^20 21^ and recently published policy briefs that help regulate genomics.^22^

## Data Availability

Data are available in a public, open access repository. All data relevant to the study are included in the article or uploaded as supplementary information. All data relevant to the study are included in the article or can be reached via the URLs provided. Relevant DOIs: repository: Zenodo licences: all are Creative Commons Attribution 4.0 International. Supplemental figure 1: https://doi.org/10.5281/zenodo.6305055. Supplemental file S1: https://doi.org/10.5281/zenodo.6305250. Supplemental file S2: https://doi.org/10.5281/zenodo.6305563.
